# Temporal Trends in Analgesic Use in Long‐Term Care Facilities: A Systematic Review of International Prescribing

**DOI:** 10.1111/jgs.15238

**Published:** 2017-12-23

**Authors:** Francesca L. La Frenais, Rachel Bedder, Victoria Vickerstaff, Patrick Stone, Elizabeth L. Sampson

**Affiliations:** ^1^ Division of Psychiatry University College London London United Kingdom; ^2^ Division of Psychiatry Marie Curie Palliative Care Research Department University College London London United Kingdom; ^3^ Institute of Cognitive Neuroscience University College London London United Kingdom; ^4^ Barnet Enfield and Haringey Mental Health Trust Liaison Team North Middlesex University Hospital London United Kingdom

**Keywords:** analgesics, pain, nursing home, dementia

## Abstract

**Objectives:**

To explore global changes in the prescription of analgesic drugs over time in the international long‐term care (LTC) population.

**Design:**

Systematic review.

**Setting:**

We included original research articles in English, published and unpublished, that included number of participants, country and year(s) of data collection, and prescription of analgesics (analgesics not otherwise specified, opioids, acetaminophen; scheduled only, or scheduled plus as needed (PRN)).

**Participants:**

LTC residents.

**Measurements:**

We searched PubMed, EMBASE, CINAHL, International Pharmaceutical Abstracts, PsycINFO, Cochrane, Web of Science, Google Scholar, using keywords for LTC facilities and analgesic medication; hand‐searched references of eligible papers; correspondence. Studies were quality rated using an adapted Newcastle‐Ottawa scale. Pearson correlation coefficients were generated between percentage of residents prescribed an analgesic and year of data collection. If available, we investigated changes in acetaminophen and opioid prescriptions.

**Results:**

Forty studies met inclusion criteria. A moderate correlation (0.59) suggested that scheduled prescription rates for analgesics have increased over time. Similar findings were reflected in scheduled prescriptions for acetaminophen and opioids. No increase was seen when analyzing scheduled plus PRN analgesics. Use of opioids (scheduled plus PRN) appears to have increased over time.

**Conclusion:**

Worldwide, use of opioids and acetaminophen has increased in LTC residents. Research is needed to explore whether this reflects appropriate pain management for LTC residents and if PRN medication is used effectively.

A long‐term care (LTC) facility is an institution providing accommodation, meals, 24‐hour staffing, and in some cases 24‐hour nursing care. In 2011, in the United States, 3.9% of individuals aged 65 and older received LTC,^1^ similar to other developed countries.^2^, ^3^

It is suggested that LTC residents are undertreated for pain^4^, ^5^, ^6^; common painful diseases affecting LTC residents include musculoskeletal disorders, cancer, pressure sores, and neuropathies.^7^, ^8^ A large European study estimated that pain affected 48.4% of LTC residents, with 12.0% reporting uncontrolled pain,^9^ consistent with other countries,^5^, ^6^ including a U.S. study that found that 23.0% of residents reporting persistent pain did not receive scheduled analgesics.^10^ Dementia is often underdiagnosed in this population^11^; cognitively impaired residents may not remember, understand, or communicate their pain, presenting a complex challenge for care staff assessing pain.^12^, ^13^ Poorly managed pain can lead to distress, poor quality of life,^14^, ^15^ worsening cognition, and depression.^16^, ^17^

Prescribers should take a stepwise approach from nonopioids, used for mild to moderate pain (e.g., acetaminophen, considered a first‐line treatment because it is well tolerated) to opioids, generally used for severe acute pain or chronic pain but with risk of side effects such as sedation, constipation, nausea, and vomiting. In older adults multimorbidity and polypharmacy increase the likelihood of adverse events.^8^, ^18^, ^19^

## Review Aims

Our aim was to investigate whether, and how, international prescribing patterns of analgesic medication for LTC residents have changed over time. Specific objectives were to explore changes in the prescription of analgesic drugs, explore changes in prescribing of opioids and acetaminophen; and examine changes in scheduled medications and scheduled plus as‐needed (pro re nata (PRN)) medications.

## Method

### Search Strategy

We used a three‐step search strategy. To refine the search terms, an initial limited search of PubMed was run, followed by analysis of the text words and Medical Subject Heading terms contained in the title, abstract, and index of identified papers. Then a search was run using identified key words and index terms (for LTC facilities and analgesics; see Appendix S1) across included databases until December 2016 (PubMed (including Medline, 1966–present), EMBASE (1947–present), CINAHL (1937–present), International Pharmaceutical Abstracts (1970–present), PsycINFO (1880s–present), Cochrane (1898–present), Web of Science (1900–present) and Google Scholar). There were no restrictions on country. Finally, references of included articles were hand searched.

### Eligibility Criteria

Original research articles reporting prescribing of analgesics in LTC facilities were included. Single case studies and studies not published in English were excluded.

### Setting

We included LTC facilities (residential homes (institution with board, meals, 24‐hour staffing), nursing homes (as before plus 24‐hour nurse coverage), group dwellings (if deemed suitable based on description)). We excluded assisted living accommodations, sheltered accommodations, retirement apartments, and hospitals.

### Study Population

Included participants were residents in an eligible setting where the majority of participants were aged 55 and older in studies that did not focus on a specific illness or condition. A study population was ineligible if it consisted of newly admitted (admission <3 months) residents; those diagnosed with a specific illness, those receiving palliative care, individuals who were included only if they were deemed to be in pain; individuals who were included only because of polypharmacy; incidence of adverse drug event; incidence of fall or recent hospital admission; if dementia or cognitive impairment were excluded; mild cognitive impairment or severe cognitive impairment only; or where residents with severe impairment were excluded, and the number of residents in the excluded population exceeded the number of included participants.

### Data

One reviewer (FL) independently screened titles, abstracts, and full‐text articles and extracted the number or percentage of residents prescribed analgesics (including analgesic‐antipyretics), opioids, or acetaminophen; the total number of participants; if available the number of LTC facilities; and year and country of data collection. Data were ineligible if prescriptions included drugs that were potentially not for analgesia (e.g., MO1 drug class) or analgesics combined with other medications, such as disease‐modifying antirheumatic products; only PRN data were available; medication was recorded only if the drug was administered within a specific time window (unless daily, when it was counted as scheduled only); or only weighted percentages were given. If authors indicated that they had collected relevant but unpublished information, they were contacted. There was no restriction on study design. Randomized controlled trials were included if baseline data were published. For longitudinal studies, data were analyzed from the first time point that was at least 3 months after admission to the LTC facility to avoid confounding variables associated with newly admitted residents.

### Data Extraction and Quality Checking

Two researchers independently extracted and reviewed data (FL, RB). Eligible studies were assessed for methodological validity using a 5‐point scale (Appendix S2) adapted from the Newcastle‐Ottawa scale^20^ and Boyle scale.^21^ Studies were deemed strong, moderate, or weak (adapted from Boyle^21^) by rating representativeness of the target cohort, adequacy and standardization of data collection tools, participation rate, and inclusion of cluster sampling in analysis. If a study did not account for cluster sampling, it was demoted by 1 quality rating. If answers were unclear, the authors were contacted. If they could not be reached, we used the lowest score for that item. Final scores were resolved through discussion and with a third independent author (ELS).

### Analysis

The percentage of residents prescribed analgesics was calculated to one decimal place. Data were specified as scheduled drugs only or scheduled plus PRN; if not explicitly mentioned, they were deemed to be scheduled plus PRN. Articles that included scheduled medications and scheduled plus PRN medications or published data from 2 time points were divided into “cohorts” for separate analysis. Analgesic medications were coded using the Anatomical Therapeutic Chemical classification system^22^ (Appendix S3).

We quantified study heterogeneity (*I* ^2^ > 75% is considerable heterogeneity). If the data were statistically viable, we planned to meta‐analyze them, but if that was not possible, we planned to generate correlation coefficients using the Pearson correlation. The Pearson correlation is sensitive to outliers, so we planned to exclude extreme outliers, identified from the scatter plot, if there was sufficient clinical justification to do so based on the original article's discussion. Stata version 14 (Stata Corp., College Station, TX) was used.

## Results

**Figure 1:**
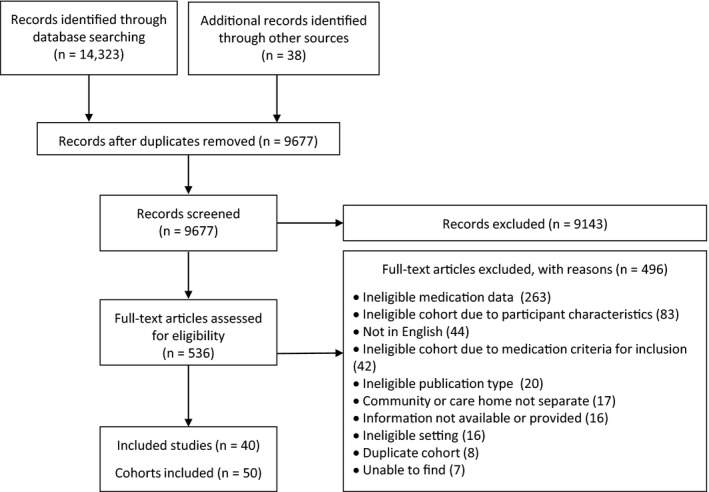
Flow diagram of study selection.

Of 14,323 citations reviewed, 40 studies were included (Figure 1). From the 40 studies, 50 cohorts were eligible. Supplementary Appendix S4 describes study characteristics and quality ratings.

Data were divided according to prescription type: scheduled only (n = 15) or scheduled plus PRN (n = 35). For scheduled only, the median number of participants per study was 551 (range 215–7,309). For scheduled plus PRN prescriptions, the median was 595 (range 13–16,126).

Data were available from 16 countries. One study included data from across Europe (excluding Italy). The countries with the most cohorts were Australia (n = 8), Norway (n = 7), and the United States (n = 6). All other cohorts were from Europe, North America, and Australia. We were unable to meta‐analyze because of heterogeneity (prescriptions of scheduled analgesics *I* ^2^ = 99.1, scheduled plus PRN analgesics, *I* ^2^ = 99.8).

### Quality Rating

Six cohorts were scored as being of strong quality, 20 as moderate, and 24 as weak. The main reasons for low scores were authors not using cluster sampling and lack of detail about data collection methods.

### Analgesics

#### Temporal Changes in Prescriptions of Scheduled Analgesics

Fifteen cohorts were eligible (Table 1) (data drawn from 17,670 residents and at least 490 LTC facilities in 8 countries). Two studies,^23^, ^24^ accounting for 7,545 residents, did not provide the number of included LTC facilities.

**Table 1:** Cohorts Included in Analysis of Scheduled Analgesic Prescribing Rates

Study	Year Data Collection Ended	Country	Residents Prescribed Regular Analgesics, % (n = 18,867)
Hoffmann and Schmiemann^26^,^a^	2015	Germany	33.7
Tan, Visvanathan^54^,^a^,^b^	2014	Australia	75.2
Bauer, Pitzer^55^,^b^	2012	Austria	52
Veal, Bereznicki^23^,^b^	2012	Australia	62.8
Sandvik, Selbaek^19^,^a^,^b^	2011	Norway	57.6
Kölzsch, Wulff^27^	2010	Germany	32
Krüger, Folkestad^56^,^b^	2008	Norway	54.8
Lövheim, Karlsson^24^,^a^,^b^	2006	Sweden, Finland	60.6
Reynolds, Hanson^32^	2004	United States	32
Sandvik, Selbaek^19^,^a^,^b^	2004	Norway	45
Decker, Culp^57^	2003	United States	45.6
Smalbrugge, Jongenelis^33^,^a^,^b^	2001	Netherlands	45.9
Sandvik, Selbaek^19^,^a^,^b^	2000	Norway	34.9
Nygaard, Naik^58^,^a^,^b^	1997	Norway	29.9
Nygaard and Naik^25^	1996	Norway	23

a Acetaminophen data available.

b Opioid data available.

**Figure 2:**
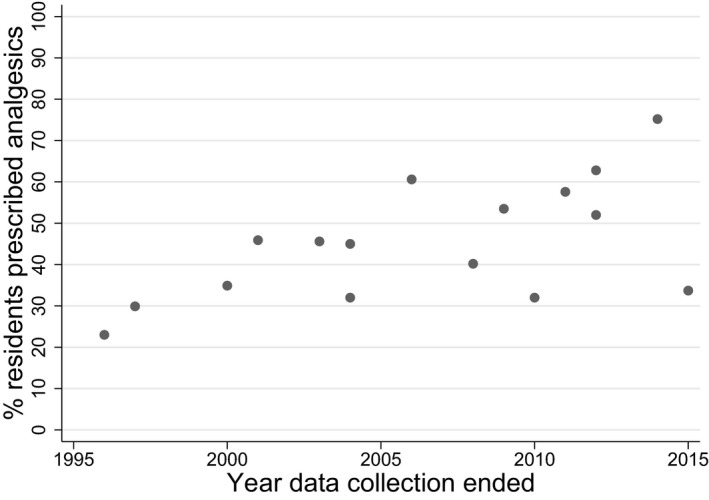
Percentage of residents prescribed scheduled analgesic medication over time.

Figure 2 suggests that, between 1996 and 2015, analgesic prescribing increased in LTC facilities. Data from a Norwegian study show that 23% of residents were prescribed scheduled analgesics in 1996, compared with 57.6% in 2011.^19^, ^25^ Two studies, both from Germany, reported lower levels: one^26^ reported that 33.7% of residents were prescribed scheduled analgesics in 2014, and another^27^ reported a 32% prescription rate in 2010. The correlation between prescription prevalence and final year of data collection was 0.59, showing a moderate positive trend.

#### Temporal Changes in Prescriptions of Scheduled Opioids and Acetaminophen

Ten studies included data on opioid prescriptions (correlation coefficients (Rs) = 0.94), and eight on acetaminophen prescriptions (Rs = 0.93, excluding one outlier that reported very low acetaminophen use (2.5%)). The number of scheduled prescriptions of opioids and acetaminophen has increased over time.

#### Temporal Changes in Prescriptions of Scheduled Plus PRN Analgesics

Thirty‐one cohorts were eligible (73,938 residents, at least 526 LTC facilities in 16 countries plus Europe, excluding Italy; Table 2). There were 10 cohorts, accounting for 46,211 residents, that did not provide the number of LTC facilities included.

**Table 2:** Cohorts Included in Analysis of Scheduled Plus As‐Needed Analgesic Prescribing Rates

Study	Year Data Collection Ended	Country	Residents Prescribed Regular Analgesics, % (n = 73,938)
Hoffmann and Schmiemann^26^,^a^	2015	Germany	73.8
Lövheim (2017, personal communication, 3 April)^a^,^b^	2013	Sweden	66.6
Onder, Vetrano^30^,^b^	2013	Europe, not including Italy	28
Onder, Vetrano^30^,^b^	2013	Italy	16
Bauer, Pitzer^55^	2012	Austria	83
Kaasalainen, Wickson‐Griffiths^59^	2012	Canada	90
Veal, Bereznicki^23^	2012	Australia	90.8
Taxis, Kochen^60^,^b^	2009	Australia, Netherlands	80.8
Boerlage, Masman^41^,^b^	2008	Netherlands	45.8
Lövheim (2017, personal communication, 3 April)^a^,^b^	2007	Sweden	62.8
Stafford, Alswayan^61^	2007	Australia	56.8
Torvik, Kaasa^62^,^b^	2006	Norway	54.7
Carey, De Wilde^63^	2005	United Kingdom	60.6
Elseviers, Vander Stichele^64^	2005	Belgium	41.5
Roughead, Gilbert^65^,^a^	2005	Australia	53.8
Reynolds, Hanson^32^	2004	United States	68.6
Bergman, Olsson^66^,^b^	2003	Sweden	61.5
Snowdon, Day^67^	2003	Australia	63.6
Jervis, Shore^28^	2002	United States	95
Smalbrugge, Jongenelis^33^	2001	Netherlands	54.5
Jyrkka, Vartiainen^68^	1998	Finland	54
King^69^,^a^,^b^	1997	Australia	74
O'Grady and Weedle^70^	1997	Ireland	20
Kaasalainen, Middleton^29^	1996	Canada	95
Neutel, Perry^71^	1996	Canada	33.5
Van Dijk, de Vries^72^,^b^	1995	Netherlands	53
King^69^,^a^,^b^	1994	Australia	60.9
Ferrell, Ferrell^73^	1990	United States	78
Vander Stichele, Mestdagh^74^	1990	Belgium	26
Williams, Nichol^31^	1990	United States	38.3
Passmore, Crawford^75^	1989	Northern Ireland	24.8
Hatton^76^	1987	England	43
Nolan and O'Malley^77^	1987	Ireland	27
Yakabowich, Keeley^78^	1987	Canada	58.5
Primrose, Capewell^79^	1984	Scotland	32

a Acetaminophen data available.

b Opioid data available.

Because the scatter plot did not suggest a trend, it was not appropriate to run a correlation. Scheduled plus PRN prescriptions have not changed since 1984. Several studies^24^, ^28^, ^29^ show very high prescribing rates (>90%). One of the most recent studies (from 2013) reported the lowest prescribing rate (16%).^30^ Of the four U.S. studies, the earliest (1990) reported that 38.3% of residents were prescribed analgesics,^31^ compared with 68.6% in 2004.^32^

#### Temporal Changes in Prescriptions of Scheduled Plus PRN Opioids and Acetaminophen

For scheduled plus PRN prescriptions for opioids and acetaminophen over time, there was a positive linear trend for opioids over time, with a moderate correlation coefficient (0.48). It appears that scheduled plus PRN prescriptions for opioids have increased. Opioids were prescribed less frequently than acetaminophen.

## Discussion

### Prescribing Patterns

We have demonstrated a multinational trend of increased prescription of scheduled analgesics, with corroborative findings for acetaminophen and opioids. Intracountry longitudinal studies (e.g., increases in Norway between 2000 and 2011) and intercountry comparisons (in 2000–01, 34.9% of Norwegian residents and 45.9% of Dutch residents were prescribed analgesics, and in 2011–12, 57.6% of Norwegian residents and 62.8% Australian residents were prescribed analgesics) support this finding.^19^, ^23^, ^33^

There does not appear to be a temporal trend for scheduled plus PRN prescribing. This may be because there is no explicit guidance regarding assessment before giving PRN medication^12^ and individual clinical preference continues to influence prescribing.

As expected, acetaminophen remained the most commonly prescribed analgesic,^4^, ^23^, ^34^ and prescriptions have increased. The exception is Germany, probably because of the frequent use of dipyrone, a drug banned in several other countries because of risk of agranulocytosis.^26^

Several factors may have influenced increases in opioid prescriptions. Clinicians are more cautious about nonsteroidal antiinflammatory drugs (NSAIDS) and may prescribe opioids as an alternative. A Finnish study saw a reduction in NSAID use in LTC facilities from 13.0% in 2003 to 2.6% in 2011,^35^ as did a Norwegian study (6.8% in 2000 to 3.2% in 2011), alongside increases in opioids and acetaminophen.^19^ Concerns have been expressed that opioids are used for their sedative effect, not just pain.^13^, ^35^ Another concern is that opioids may be wrongly prescribed for neuropathic pain, for which an adjuvant drug may be more effective; the prevalence of adjuvant drugs does not match the prevalence of neuropathic pain.^19^, ^35^

More detailed studies have identified that strong opioids are used more than weak opioids.^4^, ^19^, ^36^ The introduction of buprenorphine and fentanyl patches may have contributed to use of strong opioids.^37^ A Danish study reported that nursing home residents were more likely to receive transdermal opioids.^13^ Their use may be appealing because of ease of administration,^38^ but U.S. and U.K. guidelines advise that extended‐release opioids should not be the first choice because of negative side effects.^18^, ^38^, ^39^, ^40^

### Quality Rating

The ranges of prescribing prevalence were similar for high‐ and low‐quality studies. It is troubling that there were so few high‐quality studies (6 out of 50 cohorts). There was no clear indication that higher‐quality studies produced mutually consistent results in terms of prescribing prevalence, which may be because of the heterogeneity of samples and settings.

### Cultural Factors

Several studies found a low prevalence of analgesic use. In Italy, 24% of residents reporting pain did not receive analgesics, and authors commented that medication was neither appropriately nor effectively managing pain.^30^ A Dutch study reported that 38% of residents in “substantial” pain received no analgesics, noting that pain was not included in national nursing home performance indicators.^41^ Another study reported remarkably low analgesic use in Poland. Only 28.8% of residents received analgesics, and only 21.4% of these received scheduled pain relief. Authors commented that pain is not routinely assessed in nursing homes.^42^ Where low analgesic use is reported, authors often describe a cultural climate that does not prioritize pain assessment. In Italy, where low rates of analgesic prescriptions are reported,^4^ nonpharmacological analgesia is used more frequently, as it is in Finland.

### Limitations

Sample sizes varied greatly, from primary data collection studies involving 1 LTC facility to databases of thousands. One doctor or practice typically manages LTC prescribing, which is thus subject to individual preferences. Data from a small number of facilities may indicate less typical prescribing patterns than a larger sample and contribute to the high levels of observed heterogeneity. Conversely, it can be more difficult to ensure reliability of database records because they depend on accurate input from the LTC facility.^43^ There were no studies from South America, Africa, or Asia, and conclusions are not generalizable outside Western Europe, North America, and Australia. Lastly, it has been suggested that neuropathic pain, estimated to be present in 8% to 11% of elderly and nursing home populations,^44^, ^45^ is often treated inappropriately. This review has not explored prescriptions of neuropathic analgesics because they may be prescribed for other conditions, and most studies do not collect information on prescribing indications.

### Clinical and Policy Implications

Many countries have shifted from NSAID use, and in their place other analgesics may be prescribed. In Australia, 2005 national prescribing guidelines, which highlighted good practice in pain management in residential care,^23^, ^46^ may be influencing increasing analgesic use, and a UK increase in fentanyl use may have occurred after its licensing for noncancer pain in 2002. There has been growing interest in pain in individuals with dementia and LTC facilities highlighting undertreatment,^9^, ^12^ leading to greater use of assessment tools and treatment guidelines.^8^, ^18^, ^47^ Furthermore, there has been more research into behavioral and psychological symptoms of dementia and pain.^48^, ^49^ These studies, combined with policy pressure to limit use of psychotropics, such as the Omnibus Budget Reconciliation Act of 1987, may have contributed to the increase in analgesic prescriptions, particularly opioids.^50^, ^51^

### Future Research Needed

An increase in analgesic prescribing does not necessarily mean that residents are receiving the most appropriate treatment,^36^ and more frequent pain assessment does not necessarily equate to more analgesia.^52^ Medication is often prescribed as needed, and administration depends upon staff and their ability to assess pain accurately. This is particularly relevant for cognitively impaired residents who cannot communicate their pain; regular prescriptions may ensure that this population is at less risk of undertreatment.^53^ Research into using clinical decision‐making algorithms (with stepped treatment approaches), greater collaboration between professionals such as pharmacists and palliative care nurses, and developing interventions to empower and engage the whole care team involved in regularly assessing pain and evaluating pain management strategies could address the disconnect between recognizing and treating pain.^37^

## Conclusion

This is the first systematic review to investigate changes in prescribing patterns of analgesics in the international LTC population. We included data from all studies reporting analgesic use and demonstrated that increases in prescribing seen in smaller studies are representative of an international upward trend, providing a context for current prescribing practices in LTC facilities and insight into the influence of research focus and policy changes.

## Supporting information


**Appendix S1.** Search terms used in each database

**Appendix S2.** Adapted quality rating scale

**Appendix S3.** Anatomical Therapeutic Chemical codes used to describe analgesics included in cohorts

**Appendix S4.** Table of included cohorts: study characteristics and quality ratings
