# Supplementary material for: Temporal Trends in Analgesic Use in Long‐Term Care Facilities: A Systematic Review of International Prescribing
Source: J Am Geriatr Soc. 2017 Dec 23;66(2):376–82. doi: 10.1111/jgs.15238 (PMC5838548; doi:10.1111/jgs.15238)
Supplement: Supplementary file 1 — Appendix S1. Search terms used in each database [file JGS-66-376-s001.docx]

**Supplementary Appendix S1: Search terms used in each database**

**PubMed/Medline/International Pharmaceutical Abstracts**

| 1. exp home for the aged/ or exp elderly care/ or exp institutional care/ |  |
| --- | --- |
| 2. home for the aged.mp. or exp home for the aged/ |  |
| 3. residential facilities.mp. or exp residential home/ |  |
| 4. ("care home" or "care homes").mp. [mp=title, abstract, heading word, drug trade name, original title, device manufacturer, drug manufacturer, device trade name, keyword] |  |
| 5. ("long term care" or "long-term care" or "longterm care").mp. [mp=title, abstract, heading word, drug trade name, original title, device manufacturer, drug manufacturer, device trade name, keyword] |  |
| 6. ("aged care" or "aged-care").mp. [mp=title, abstract, heading word, drug trade name, original title, device manufacturer, drug manufacturer, device trade name, keyword] |  |
| 7. ("residential home" or "residential homes").mp. [mp=title, abstract, heading word, drug trade name, original title, device manufacturer, drug manufacturer, device trade name, keyword] |  |
| 8. "assisted living".mp. or exp assisted living facility/ |  |
| 9. (convalescent or elderly or geriatric or aged or nursing or residential or care or healthcare).mp. [mp=title, abstract, heading word, drug trade name, original title, device manufacturer, drug manufacturer, device trade name, keyword] |  |
| 10. (home or facility or centre or center or facilities).mp. [mp=title, abstract, heading word, drug trade name, original title, device manufacturer, drug manufacturer, device trade name, keyword] |  |
| 11. ((convalescent or elderly or geriatric or aged or nursing or residential or care or healthcare) adj (home or facility or centre or center or facilities)).mp. |  |
| 12. analgesic.mp. or exp analgesic agent/ |  |
| 13. pain management.mp. or exp analgesia/ |  |
| 14. 12 or 13 |  |
| 15. ("nursing home" or "nursing homes").mp. [mp=title, abstract, heading word, drug trade name, original title, device manufacturer, drug manufacturer, device trade name, keyword] |  |
| 16. 1 or 2 or 3 or 4 or 5 or 6 or 7 or 8 or 11 or 15  17. 14 and 16 |  |

**Embase**

| 1. exp home for the aged/ or exp elderly care/ or exp institutional care/ |  |
| --- | --- |
| 2. home for the aged.mp. or exp home for the aged/ |  |
| 3. residential facilities.mp. or exp residential home/ |  |
| 4. ("aged care" or "aged-care").mp. [mp=title, abstract, heading word, drug trade name, original title, device manufacturer, drug manufacturer, device trade name, keyword] |  |
| 5. "assisted living".mp. or exp assisted living facility/ |  |
| 6. (convalescent or elderly or geriatric or aged or nursing or residential or care or healthcare).mp. [mp=title, abstract, heading word, drug trade name, original title, device manufacturer, drug manufacturer, device trade name, keyword] |  |
| 7. analgesic.mp. or exp analgesic agent/ |  |
| 8. pain management.mp. or exp analgesia/ |  |
| 9. 7 or 8 |  |
| 10. (home or homes or facility or centre or center or facilities).mp. [mp=title, abstract, heading word, drug trade name, original title, device manufacturer, drug manufacturer, device trade name, keyword] |  |
| 11. ((convalescent or elderly or geriatric or aged or nursing or residential or care or healthcare) adj (home or homes or facility or centre or center or facilities)).mp. |  |
| 12. 1 or 2 or 3 or 4 or 5 or 11  13. 9 and 12 |  |

**PsycINFO**

| 1. nursing homes/ or exp residential care institutions/ |  |
| --- | --- |
| 2. residential care institutions/ or exp nursing homes/ |  |
| 3. institutional care.mp. |  |
| 4. long term care.mp. |  |
| 5. home for the aged.mp. |  |
| 6. care home.mp. |  |
| 7. (convalescent or elderly or geriatric or aged or nursing or residential).mp. [mp=title, abstract, heading word, table of contents, key concepts, original title, tests & measures] |  |
| 8. (home or homes or facility or facilities or centre or center).mp. [mp=title, abstract, heading word, table of contents, key concepts, original title, tests & measures] |  |
| 9. ((convalescent or elderly or geriatric or aged or nursing or residential) adj (home or homes or facility or facilities or centre or center)).mp. |  |
| 10. 1 or 2 or 3 or 4 or 5 or 6 or 9 |  |
| 11. exp analgesic drugs/ |  |
| 12. exp pain management/ |  |
| 13. exp prescription drugs/ |  |
| 14. 11 or 12 or 13 |  |

15. 10 and 14

**Cochrane**

1. MeSH descriptor: [Analgesics] explode all trees
2. MeSH descriptor: [Pain Management] explode all trees
3. MeSH descriptor: [Prescription Drugs] explode all trees
4. #1 or #2 or #3
5. MeSH descriptor: [Homes for the Aged] explode all trees
6. MeSH descriptor: [Nursing Homes] explode all trees
7. MeSH descriptor: [Long-Term Care] explode all trees
8. MeSH descriptor: [Residential Facilities] explode all trees
9. "care home" or "care homes"
10. "longterm care" or "long term care"
11. "aged care" or "aged-care"
12. #12 "nursing home"
13. #13 "residential home" or "residential homes"
14. #14 "assisted living"
15. #15 convalescent or elderly or geriatric or aged or nursing or residential or care or healthcare
16. #16 home or facility or centre or center or facilities
17. #17 #15 adj #16
18. #18 #17 or #14 or #13 or #12 or #11 or #10 or #9 or #8 or #7 or #6 or #5
19. #19 #18 and #4

**Web of Science**

1. **TOPIC:** ("home for the aged" or "institutional care" or "care home") *OR*
2. **TOPIC:** ((convalescent or elderly or geriatric or aged or nursing or residential) AND (home or homes or facility or facilities)) *AND*
3. **TOPIC:** (analges* or "analgesic agent" or "pain management" or analgesic) *AND*
4. **TOPIC:** (resident)

**CINAHL**

1. MH nursing homes or residential care
2. nursing home
3. nursing homes or long-term facilities
4. MH nursing homes or nursing home patients
5. nursing homes or housing for the elderly or long term care
6. (MH "Nursing Homes+") OR (MH "Nursing Home Patients")
7. (MH "Long Term Care")
8. "home for the aged"
9. (MH "Institutionalization") OR "institutional care"
10. "aged-care"
11. convalescent or elderly or geriatric or aged or nursing or residential
12. "care home" or "care homes"
13. home or homes or facility or facilities or centre or center
14. S11 adj S13
15. S1 OR S2 OR S3 OR S5 OR S6 OR S7 OR S8 OR S9 OR S10 OR S12 OR S14
16. (MH "Analgesics+")
17. "analgesic agent"
18. "pain management"
19. (MH "Drugs, Prescription")
20. (MH "Prescriptions, Drug")
21. S17 OR S18 OR S19 OR S20
22. S15 AND S21

**Google Scholar**

(prescription* or prescribing or drug* or medicine* or medication* or pharma* or polypharmacy) and (residential or care home* or care facilit* or nursing home*)
