# Supplementary material for: Temporal Trends in Analgesic Use in Long‐Term Care Facilities: A Systematic Review of International Prescribing
Source: J Am Geriatr Soc. 2017 Dec 23;66(2):376–82. doi: 10.1111/jgs.15238 (PMC5838548; doi:10.1111/jgs.15238)
Supplement: Supplementary file 2 — Appendix S2. Adapted quality rating scale [file JGS-66-376-s002.doc]

**Supplementary Appendix S2: Adapted quality rating scale**

**STUDY:**

**1) Representativeness of the target cohort**

a) truly representative of the average LTC resident

b) somewhat representative of the average LTC resident

c) selected group of users e.g. nurses, volunteers, veterans

d) no description of the derivation of the cohort

**2) Were data collection tools adequate?** (analgesic prescription information)

a) Yes – *medical records, insurance data*

b) No – *nurse or self report*

c) Can’t tell

**3) Were data collection tools standardised?** (analgesic prescription information)

a) Yes

b) No

c) Can’t tell

**4) What percentage of selected individuals agreed to participate?**

a) 60-100% agreement

b) Less than 60% agreement

c) Can’t tell

**5) Were special features of the sampling design accounted for in the analysis?**

a) Yes/not applicable

b) No

**STRONG:** 1 = a; and 2 = a; and 3 = a; and 4 = a; and 5 = a

**MODERATE:** 1 = a/b; and 2 = a; and 3 = a; and 4 = b/c

**WEAK:** 1 = c/d; or 2 = b/c; or 3 = b/c or 4 = c

*If 5) = b) No then decrease by one level (i.e. Strong>moderate, moderate>weak)*

**RATER 1: RATER 2:**

**Is there a discrepancy? Yes No FINAL RATING:**

**If yes, why?** Differences in interpretation of criteria Oversight

Differences in interpretation of study Other
