# Supplementary material for: Temporal Trends in Analgesic Use in Long‐Term Care Facilities: A Systematic Review of International Prescribing
Source: J Am Geriatr Soc. 2017 Dec 23;66(2):376–82. doi: 10.1111/jgs.15238 (PMC5838548; doi:10.1111/jgs.15238)
Supplement: Supplementary file 3 — Appendix S3. Anatomical Therapeutic Chemical codes used to describe analgesics included in cohorts [file JGS-66-376-s003.docx]

Supplementary table S3. ATC codes used to describe analgesics included in cohorts

| N02 | General analgesics and antipyretics |
| --- | --- |
| N02A | Opioids |
| N02B | Other analgesics and antipyretics |
| N02BE01 | Acetaminophen |
| M01 | Anti-inflammatory and anti-rheumatic products |
| M01A | Anti-inflammatory and anti-rheumatic products, non-steroids |
| M02A | Topical products for joint and muscular pain |
| B01AC06 | Acetylsalicylic acid (aspirin) |
