# Supplementary material for: Temporal Trends in Analgesic Use in Long‐Term Care Facilities: A Systematic Review of International Prescribing
Source: J Am Geriatr Soc. 2017 Dec 23;66(2):376–82. doi: 10.1111/jgs.15238 (PMC5838548; doi:10.1111/jgs.15238)
Supplement: Supplementary file 4 — Appendix S4. Table of included cohorts: study characteristics and quality ratings [file JGS-66-376-s004.docx]

Supplementary Appendix S4. Table of included cohorts: study characteristics and quality ratings

| **Included cohorts –**  **Author and year of publication** | **Country** | **Year data collection ended** | **n** | **Number of LTC facilities** | **Quality rating** | **Regular prescriptions only, or regular + PRN** |
| --- | --- | --- | --- | --- | --- | --- |
| Bauer, Pitzer, Schreier, Osterbrink, Alzner, Iglseder ^1^ | Austria | 2012 | 425 | 12 | weak | both |
| Bauer, Pitzer, Schreier, Osterbrink, Alzner, Iglseder ^1^ | Austria | 2012 | 425 | 12 | weak | regular |
| Bergman, Olsson, Carlsten, Waern, Fastbom ^2^ | Sweden | 2003 | 7904 | nk | moderate | both |
| Boerlage, Masman, Tibboel, Baar, Van Dijk ^3^ | Netherlands | 2008 | 201 | 1 | strong | both |
| Carey, De Wilde, Harris et al. ^4^ | UK | 2005 | 2864 | nk | moderate | both |
| Decker, Culp, Cacchione ^5^ | US | 2003 | 215 | 13 | weak | regular |
| Elseviers, Vander Stichele, Van Bortel ^6^ | Belgium | 2005 | 2510 | 76 | weak | both |
| Ferrell, Ferrell, Osterweil ^7^ | US | 1990 | 92 | 1 | moderate | both |
| Hatton ^8^ | England | 1987 | 449 | 25 | weak | both |
| Hoffmann, Schmiemann ^9^ | Germany | 2015 | 852 | 21 | strong | regular |
| Hoffmann, Schmiemann ^9^ | Germany | 2015 | 852 | 21 | strong | both |
| Jervis, Shore, Hutt, Manson ^10^ | US | 2002 | 45 | 1 | moderate | both |
| Jyrkka, Vartiainen, Hartikainen, Sulkava, Enlund ^11^ | Finland | 1998 | 13 | nk | moderate | both |
| Kaasalainen, Middleton, Knezacek et al. ^12^ | Canada | 1996 | 83 | 1 | moderate | both |
| Kaasalainen, Wickson-Griffiths, Akhtar-Danesh et al. ^13^ | Canada | 2012 | 345 | 6 | weak | both |
| King ^14^ | Australia | 1994 | 998 | 15 | moderate | both |
| King ^14^ | Australia | 1997 | 414 | 11 | weak | both |
| Kölzsch, Wulff, Ellert et al. ^15^ | Germany | 2010 | 560 | 40 | weak | regular |
| Krüger, Folkestad, Geitung, Eide, Grimsmo ^16^ | Norway | 2008 | 513 | 7 | moderate | regular |
| Hemmingsson, Gustafsson, Isaksson et al. ^17^ | Sweden | 2013 | 1849 | nk | weak | both |
| Hemmingsson, Gustafsson, Isaksson, et al. ^17^ | Sweden | 2007 | 2764 | nk | weak | both |
| Lövheim, Karlsson, Gustafson ^18^ | Sweden, Finland | 2006 | 236 | nk | weak | regular |
| Neutel, Perry, Maxwell ^19^ | Canada | 1996 | 227 | 1 | strong | both |
| Nolan, O'Malley ^20^ | Ireland | 1987 | 301 | 11 | moderate | both |
| Nygaard, Naik ^21^ | Norway | 1996 | 347 | 15 | weak | regular |
| Nygaard, Naik, Ruths, Straand ^22^ | Norway | 1997 | 1042 | 15 | moderate | regular |
| O'Grady, Weedle ^23^ | Ireland | 1997 | 115 | 1 | weak | both |
| Onder, Vetrano, Cherubini et al. ^24^ | Italy | 2013 | 3179 | nk | weak | both |
| Onder, Vetrano, Cherubini, et al. ^24^ | Europe not including Italy | 2013 | 3608 | nk | weak | both |
| Passmore, Crawford, Beringer, Gilmore, Montgomery ^25^ | N. Ireland | 1989 | 595 | nk | moderate | both |
| Primrose, Capewell, Simpson, Smith ^26^ | Scotland | 1984 | 400 | 18 | weak | both |
| Reynolds, Hanson, DeVellis, Henderson, Steinhauser ^27^ | US | 2004 | 551 | 6 | weak | both |
| Reynolds, Hanson, DeVellis, Henderson, Steinhauser ^27^ | US | 2004 | 551 | 6 | weak | regular |
| Roughead, Gilbert, Woodward ^28^ | Australia | 2005 | 16126 | nk | weak | both |
| Sandvik, Selbaek, Kirkevold, Husebo, Aarsland ^29^ | Norway | 2004 | 1163 | 26 | strong | regular |
| Sandvik, Selbaek, Kirkevold, Husebo, Aarsland ^29^ | Norway | 2011 | 1858 | 64 | strong | regular |
| Sandvik, Selbaek, Kirkevold, Husebo, Aarsland ^29^ | Norway | 2000 | 1926 | 251 | weak | regular |
| Smalbrugge, Jongenelis, Pot, Beekman, Eefsting ^30^ | Netherlands | 2001 | 290 | 14 | weak | regular |
| Smalbrugge, Jongenelis, Pot, Beekman, Eefsting ^30^ | Netherlands | 2001 | 290 | 14 | weak | both |
| Snowdon, Day, Baker ^31^ | Australia | 2003 | 3054 | 50 | moderate | both |
| Stafford, Alswayan, Tenni ^32^ | Australia | 2007 | 2345 | 41 | moderate | both |
| Tan, Visvanathan, Hilmer et al. ^33^ | Australia | 2014 | 383 | 6 | moderate | regular |
| Taxis, Kochen, Wouters et al. ^34^ | Australia, Netherlands | 2009 | 3597 | 32 | moderate | both |
| Torvik, Kaasa, Kirkevold, Rustøen ^35^. | Norway | 2006 | 214 | 7 | moderate | both |
| Van Dijk, de Vries, Van den Berg, Brouwers, De Jong-van den Berg ^36^ | Netherlands | 1995 | 2355 | 6 | moderate | both |
| Vander Stichele, Mestdagh, Van Haecht, De Potter, Bogaert ^37^ | Belgium | 1990 | 198 | 20 | weak | both |
| Veal, Bereznicki, Thompson, Peterson ^38^ | Australia | 2012 | 7309 | nk | moderate | both |
| Veal, Bereznicki, Thompson, Peterson ^38^ | Australia | 2012 | 7309 | nk | moderate | regular |
| Williams, Nichol, Lowe, Yoon, McCombs, Margolies ^39^ | US | 1990 | 818 | 61 | weak | both |
| Yakabowich, Keeley, Montgomery ^40^ | Canada | 1987 | 6848 | 88 | moderate | both |

*nk = data not known

**Study published data on at least two eligible cohorts

1. Bauer U, Pitzer S, Schreier MM, Osterbrink J, Alzner R, Iglseder B. Pain treatment for nursing home residents differs according to cognitive state - a cross-sectional study. *BMC Geriatr.* 2016;16:124.

2. Bergman Å, Olsson J, Carlsten A, Waern M, Fastbom J. Evaluation of the quality of drug therapy among elderly patients in nursing homes: A computerized pharmacy register analysis. *Scandinavian journal of primary health care.* 2007;25(1):9-14.

3. Boerlage A, Masman A, Tibboel D, Baar F, Van Dijk M. Is pain measurement a feasible performance indicator for Dutch nursing homes? A cross-sectional approach. *Pain Manag Nurs.* 2013;14(1):36-40.

4. Carey IM, De Wilde S, Harris T, et al. What factors predict potentially inappropriate primary care prescribing in older people? *Drugs & aging.* 2008;25(8):693-706.

5. Decker SA, Culp KR, Cacchione PZ. Evaluation of musculoskeletal pain management practices in rural nursing homes compared with evidence-based criteria. *Pain Management Nursing.* 2009;10(2):58-64.

6. Elseviers MM, Vander Stichele RR, Van Bortel L. Drug utilization in Belgian nursing homes: impact of residents' and institutional characteristics. *Pharmacoepidemiology and drug safety.* 2010;19(10):1041-1048.

7. Ferrell BA, Ferrell BR, Osterweil D. Pain in the nursing home. *Journal of the American Geriatrics Society.* 1990;38(4):409-414.

8. Hatton P. "Primum non nocere" - an analysis of drugs prescribed to elderly patients in private nursing homes registered with Harrogate Health Authority. *Care of the Elderly.* 1990;2(4):166-169.

9. Hoffmann F, Schmiemann G. Pain medication in German nursing homes: a whole lot of metamizole. *Pharmacoepidemiol Drug Saf.* 2016;25(6):646-651.

10. Jervis LL, Shore J, Hutt E, Manson SM. Suboptimal pharmacotherapy in a tribal nursing home. *Journal of the American Medical Directors Association.* 2007;8(1):1-7.

11. Jyrkka J, Vartiainen L, Hartikainen S, Sulkava R, Enlund H. Increasing use of medicines in elderly persons: A five-year follow-up of the Kuopio 75+Study. *European journal of clinical pharmacology.* 2006;62 (2):151-158.

12. Kaasalainen S, Middleton J, Knezacek S, et al. Pain and cognitive status in the institutionalized elderly: perceptions & interventions. *Journal of gerontological nursing.* 1998;24 (8):24-31; quiz 50-51.

13. Kaasalainen S, Wickson-Griffiths A, Akhtar-Danesh N, et al. The effectiveness of a nurse practitioner-led pain management team in long-term care: A mixed methods study. *Int J Nurs Stud.* 2016;62:156-167.

14. King MA. *Medication care: Databases, drug use and outcomes.* [Doctoral]. Australia: Thesis, University of Queensland; 2003.

15. Kölzsch M, Wulff I, Ellert S, et al. Deficits in pain treatment in nursing homes in Germany: A cross‐sectional study. *European Journal of Pain.* 2012;16(3):439-446.

16. Krüger K, Folkestad M, Geitung J-T, Eide GE, Grimsmo A. Psychoactive drugs in seven nursing homes. *Primary health care research & development.* 2012;13(03):244-254.

17. Hemmingsson ES, Gustafsson M, Isaksson U, et al. Prevalence of pain and pharmacological pain treatment among old people living in institutional geriatric care in 2007 and 2013. *Submitted manuscript.*

18. Lövheim H, Karlsson S, Gustafson Y. The use of central nervous system drugs and analgesics among very old people with and without dementia. *Pharmacoepidemiology and drug safety.* 2008;17(9):912-918.

19. Neutel CI, Perry S, Maxwell C. Medication use and risk of falls. *Pharmacoepidemiology and drug safety.* 2002;11(2):97-104.

20. Nolan L, O'Malley K. The need for a more rational approach to drug prescribing for elderly people in nursing homes. *Age and ageing.* 1989;18(1):52-56.

21. Nygaard H, Naik M. Drug use in homes for the aged. A comparison between mentally intact and mentally impaired residents. *Aging (Milan, Italy).* 1999;11(3):186-193.

22. Nygaard HA, Naik M, Ruths S, Straand J. Nursing-home residents and their drug use: a comparison between mentally intact and mentally impaired residents. *European journal of clinical pharmacology.* 2003;59(5-6):463-469.

23. O'Grady M, Weedle P. A descriptive study of drug therapy and cost for elderly residents in a nursing home. *Irish medical journal.* 1997;91(5):172-174.

24. Onder G, Vetrano DL, Cherubini A, et al. Prescription drug use among older adults in Italy: a country-wide perspective. *J Am Med Dir Assoc.* 2014;15(7):531 e511-535.

25. Passmore A, Crawford V, Beringer T, Gilmore D, Montgomery A. Determinants of drug utilization in an elderly population in North and West Belfast. *Pharmacoepidemiology and Drug Safety.* 1995;4(3):147-160.

26. Primrose WR, Capewell AE, Simpson GK, Smith RG. Prescribing patterns observed in registered nursing homes and long-stay geriatric wards. *Age and ageing.* 1987;16(1):25-28.

27. Reynolds KS, Hanson LC, DeVellis RF, Henderson M, Steinhauser KE. Disparities in pain management between cognitively intact and cognitively impaired nursing home residents. *Journal of pain and symptom management.* 2008;35(4):388-396.

28. Roughead EE, Gilbert AL, Woodward MC. Medication Use by Australian War Veterans in Residential Aged‐Care Facilities. *Journal of Pharmacy Practice and Research.* 2008;38(1):14-18.

29. Sandvik R, Selbaek G, Kirkevold O, Husebo BS, Aarsland D. Analgesic prescribing patterns in Norwegian nursing homes from 2000 to 2011: trend analyses of four data samples. *Age and Ageing.* 2016;45(1):54-60.

30. Smalbrugge M, Jongenelis LK, Pot AM, Beekman AT, Eefsting JA. Pain among nursing home patients in the Netherlands: prevalence, course, clinical correlates, recognition and analgesic treatment–an observational cohort study. *BMC geriatrics.* 2007;7(1):1.

31. Snowdon J, Day S, Baker W. Audits of medication use in Sydney nursing homes. *Age and ageing.* 2006;35(4):403-408.

32. Stafford AC, Alswayan MS, Tenni PC. Inappropriate prescribing in older residents of Australian care homes. *Journal of clinical pharmacy and therapeutics.* 2011;36(1):33-44.

33. Tan EC, Visvanathan R, Hilmer SN, et al. Analgesic Use and Daytime Sleepiness in Residents With and Without Dementia in Residential Aged Care Facilities. *Drugs Aging.* 2015;32(12):1045-1053.

34. Taxis K, Kochen S, Wouters H, et al. Cross-national comparison of medication use in Australian and Dutch nursing homes. *Age Ageing.* 2016.

35. Torvik K, Kaasa S, Kirkevold Ø, Rustøen T. Pain in patients living in Norwegian nursing homes. *Palliative medicine.* 2008.

36. Van Dijk K, de Vries CS, Van den Berg P, Brouwers JJ, De Jong-van den Berg L. Drug utilisation in Dutch nursing homes. *European journal of clinical pharmacology.* 2000;55(10):765-771.

37. Vander Stichele R, Mestdagh J, Van Haecht C, De Potter B, Bogaert M. Medication utilization and patient information in homes for the aged. *European journal of clinical pharmacology.* 1992;43(3):319-321.

38. Veal FC, Bereznicki LR, Thompson AJ, Peterson GM. Pharmacological management of pain in Australian aged care facilities. *Age and ageing.* 2014;43 (6):851-856.

39. Williams BR, Nichol MB, Lowe B, Yoon PS, McCombs JS, Margolies J. Medication use in residential care facilities for the elderly. *Annals of Pharmacotherapy.* 1999;33(2):149-155.

40. Yakabowich MR, Keeley G, Montgomery PR. Impact of a formulary on personal care homes in Manitoba. *Cmaj.* 1994;150 (10):1601-1610.
